# Editorial: Enhanced GNSS-based localization solutions with artificial intelligence

**DOI:** 10.3389/frobt.2023.1265989

**Published:** 2023-09-08

**Authors:** Juliette Marais, Omar García Crespillo, Li-Ta Hsu

**Affiliations:** ^1^ University Gustave Eiffel, COSYS-LEOST, Villeneuve d’Ascq, France; ^2^ Navigation Department, Institute of Communication and Navigation, German Aerospace Center (DLR), Weßling, Germany; ^3^ Department of Aeronautical and Aviation Engineering, The Hong Kong Polytechnic University, Kowloon, Hong Kong SAR, China

**Keywords:** GNSS, artificial intelligence, localization, NLOS, multipath, navigation

To answer the main challenges of sustainable development, transport systems need to change towards greener solutions, as well as be reliable and smart. This leads to the development of solutions, such as autonomous vehicles. These new systems rely on different smart sensors, among which global navigation satellite system (GNSS) receivers are considered a core element. GNSS can potentially offer global continuous positioning but can suffer from errors and may contain the presence of multiple threats at the system and local user levels. Moreover, degraded performances are expected in urban areas due to the presence of obstacles in the vicinity of the vehicle that reflect or diffract the signals and impact the time of arrival measurement. This, therefore, compromises global positioning performance requirements such as accuracy, availability, and integrity. Having good knowledge of these effects can help increase these performances at the GNSS level and also help in the development of fail-safe multi-sensor solutions as requested by most mobile users (road, railway, UAVs, etc.).

These effects can be modeled by statistical or deterministic tools but the fact is that real behavior in a real urban environment is extremely complex to model as it depends on a large number of parameters that make it difficult to recreate and completely model. With the development of artificial intelligence for data modeling and classification come new opportunities for GNSS service provision and localization evaluation or enhancement. The goal of this Research Topic of articles is to merge the different contributions using artificial intelligence in GNSS-based localization, which will improve the knowledge, characterization, and performance of such systems.

Four articles were selected for publication and, here, we provide a brief overview of each article.

Three of the contributions show an interest in using machine learning for satellite visibility, satellite state of reception definition, or multipath detection. Three of the published research papers address these detections. Garcia Crespillo et al. and Ozeki et al. focus on the detection of non-line of sight (NLOS) signals, whereas Guillard et al. focus on large multipath detection.

Various methods for the classification of LOS and NLOS signals have already been proposed, such as C/N0, using three-dimensional maps, fish-eye view, and GNSS/inertial navigation system integration. Multipath detection based on machine learning enlarges the portfolio. In “GNSS NLOS Signal Classification Based on Machine Learning and Pseudorange Residual Check,” Ozeki et al. propose a method for detecting NLOS signals that rely on a support vector machine (SVM) classifier modeled with unique features that are calculated by receiver-independent exchange format-based information and GNSS pseudo-range residual check. An NLOS-exclusion policy is then applied to a differential GNSS least-square estimation method to evaluate the impact on the position error.

Garcia Crespillo et al., in the paper titled “Robust design of a machine learning-based GNSS NLOS detector with multi-frequency features,” question how to avoid biased estimation results and guarantee generalized algorithms for different scenarios, receivers, antennas, and their specific installations and configurations, and propose new options by means of a pre-normalization of features with models extracted in open-sky (nominal) scenarios. The second main contribution focused on designing a branched (or parallel) machine learning process to handle the intermittent presence of GNSS features in certain frequencies. This allows the exploitation of measurements in all available frequencies as compared to current approaches in the literature, which are based only on single-frequency features.

In their article, “Using convolutional neural networks to detect GNSS multipath,” Guillard et al. concentrate on large multipath detection, considering that a line-of-sight (LOS) signal can be received with one or multiple reflections of the LOS signal, and propose a method to detect LOS reflections and then exclude them in real signals (Galileo E1-B and GPS L1 C/A) using only GNSS data. A conventional neural network (CNN) is developed based on inputs that represent correlator output values as a function of their delay (with respect to the prompt correlator) and as a function of time yielding a 2D image. The authors also present an exclusion policy but discuss the exclusion criterion and threshold.

Finally, the research paper titled “Improved weighting in particle filters applied to precise state estimation in GNSS,” by Zocca et al., concerns particle filters (PF) that can be used to overcome the theoretical weaknesses of the more popular Kalman filters (KFs) when the application relies on non-linear measurements models and non-Gaussian measurements errors but at the cost of a non-negligible computational complexity. Often used to infer the user position in GNSS applications, PF has also been historically recognized as a tool for artificial intelligence. Zocca et al. present a technique, named multiple weighting (MW), that reduces its computational burden by considering the information diversity provided by the input measurements about the unknown state.

The editors thank all the authors, reviewers, and the whole editorial team for the work performed to propose and finalize this Research Topic.

